# Impact of extracellular matrix stiffness on genomic heterogeneity in *MYCN*-amplified neuroblastoma cell line

**DOI:** 10.1186/s13046-020-01729-1

**Published:** 2020-10-28

**Authors:** Amparo López-Carrasco, Susana Martín-Vañó, Rebeca Burgos-Panadero, Ezequiel Monferrer, Ana P. Berbegall, Beatriz Fernández-Blanco, Samuel Navarro, Rosa Noguera

**Affiliations:** 1 Department of Pathology, Medical School, University of Valencia/INCLIVA, Valencia, Spain; 2 CIBERONC, Madrid, Spain

**Keywords:** Biotensegrity, Clonal selection, Stiffness, Vitronectin, Xenograft, 3D-bioprinting

## Abstract

**Background:**

Increased tissue stiffness is a common feature of malignant solid tumors, often associated with metastasis and poor patient outcomes. Vitronectin, as an extracellular matrix anchorage glycoprotein related to a stiff matrix, is present in a particularly increased quantity and specific distribution in high-risk neuroblastoma. Furthermore, as cells can sense and transform the proprieties of the extracellular matrix into chemical signals through mechanotransduction, genotypic changes related to stiffness are possible.

**Methods:**

We applied high density SNPa and NGS techniques to in vivo and in vitro models (orthotropic xenograft vitronectin knock-out mice and 3D bioprinted hydrogels with different stiffness) using two representative neuroblastoma cell lines (the *MYCN*-amplified SK-N-BE(2) and the *ALK*-mutated SH-SY5Y), to discern how tumor genomics patterns and clonal heterogeneity of the two cell lines are affected.

**Results:**

We describe a remarkable subclonal selection of genomic aberrations in SK-N-BE(2) cells grown in knock-out vitronectin xenograft mice that also emerged when cultured for long times in stiff hydrogels. In particular, we detected an enlarged subclonal cell population with chromosome 9 aberrations in both models. Similar abnormalities were found in human high-risk neuroblastoma with *MYCN* amplification. The genomics of the SH-SY5Y cell line remained stable when cultured in both models.

**Conclusions:**

Focus on heterogeneous intratumor segmental chromosome aberrations and mutations, as a mirror image of tumor microenvironment, is a vital area of future research.

## Background

Tumors are not homogeneous structures, but rather highly complex tissues involving many cell types, such as tumor, stromal and inflammatory cells [1–3]. Cells are primarily supported in solid tumors by the extracellular matrix (ECM), a three-dimensional (3D) dynamic network composed mainly of fibrous proteins, glycoproteins and proteoglycans [1, 4]. In tumors, cells respond to the biochemical signals of their ECM, but also to physical forces such as tension (traction and compression forces that maintain their stability), known as biotensegrity [2]. In fact, increased stromal stiffness is a classic hallmark of cancer [1, 5], as is transformation of this stiffness into chemical signals through mechanotransduction for cell advantage [5–7]. Data suggest that an aberrant ECM may promote genetic instability and can even compromise DNA repair pathways necessary to prevent malignant transformation [6, 8–10]. Among the cell population trying to respond to ECM stiffness, only the most genotypically and phenotypically adapted ones will succeed in invading new metastatic niches [11, 12]. The most advantageous cells will also segregate anchor proteins and transform the ECM into a more biochemically and tensegrally favorable microenvironment, which will also promote growth and migration [6, 13]. A continuous interplay therefore exists between tumor ECM and cell genomics and behavior [13].

ECM stiffness and composition are currently of such importance that, together with mechanotransduction, are considered promising therapeutic targets in many cancer types [14–16]. This is also the case of neuroblastoma (NB), a peripheral sympathetic tumor that causes 15% of childhood deaths from cancer [17]. We previously defined an aggressive pattern of rigid ECMs in high-risk NB (HR-NB)[18]. NB stiff ECM is rich in cross-linked collagen III fibers, poor in glycosaminoglycans, supporting sinusoidal vascular structures (blood and lymphatic) and with a high quantity of territorial vitronectin (VN, located in the cytoplasmic compartment and in a thin layer around the tumor cells) [19–22]. NB stiff ECM is also rich in collagen I fibers and fibronectin but in lesser amounts and with a more physiological-like topology than collagen III and VN [18, 23]. Moreover, VN is a glycoprotein containing multiple cell receptor binding sites including integrins, uPAR and PAI-1 [24], and which can therefore form transitory ECM element-cell junctions. It seems to anchor to ECM fibers and proteoglycans, and also disrupt cell adhesion, promoting spread and metastasis in NB [22, 25]. Some studies have shown an important role for VN in initiation and progression of hepatic [26], breast [27] and lung [28] cancers, and its particular involvement in migration in ovarian cancer [29, 30]. In a previous report we used inoculation of the NB cell lines studied here into the adrenal gland in VN knockout mice (VN-KO) to study the systemic role of VN in tumor growth. Interestingly, we found VN-dense cytoplasmic expression in tumor cells from both VN-KO and wild type (VN-WT) mice, with no differences in VN staining pattern or in tumor growth rate in early passages [22]. However, in this study we sought to resolve the particular question of whether host lack of VN has any impact on tumor genomics.

Besides *MYCN* amplification (MNA), segmental chromosomal aberrations (SCAs), *ALK* mutation and amplification, *TERT* rearrangement and *ATRX* mutation and deletion are also genetic characteristics of HR-NB [31, 32]. In HR-NB patients these genomic aberrations usually show high intratumor heterogeneity, resulting in resistance to treatment and metastasis [33–35]. We have reported a link between a SCA (1p chromosome arm deletion, 1p-) and reduction of glycosaminoglycans in the ECM of HR-NB [36]. Nevertheless, the association between other ECM features and HR-NB genomic aberrations, and clonal selection, is a neglected area of study.

3D cultures are emerging as good models for ECM-related biological studies, also reducing use of animal models. These scaffolds can reproduce complex ECM features absent from traditional, 2D or monolayer cultures, and allow us to add degrees of complexity to delve deeper into the physiopathological tumor microenvironment [37, 38]. We recently published a study using 3D bioprinted hydrogels composed of methacrylated gelatin (GelMA) and increasing concentrations of methacrylated alginate (AlgMA) to recreate different ECM stiffness. We described a pattern of aggressive behavior in NB cells cultivated for long times in rigid hydrogels [39]; however, that study did not analyze how scaffolding stiffness and growing time affected genomic heterogeneity.

In this work the described models (xenograft VN-KO mice and 3D bioprinted hydrogels) are used to discern how tumor genomics patterns and clonal heterogeneity in two NB cell lines are affected by the VN host status and tumor microenvironment stiffness, and also to extrapolate the results to primary human HR-NB. This study shows that tumor genomics and clonal genetic heterogeneity are directly related to tumor surroundings.

## Methods

### Cell culture

SK-N-BE(2), SH-SY5Y NB cell lines and SW10 mouse Schwann cell line were acquired from American Type Culture Collection (ATCC, Masassas, VA, USA). SK-N-BE(2) and SH-SY5Y cell lines were expanded in supplemented IMDM medium (Gibco, Thermofisher), and SW10 in supplemented DMEM (Gibco, Thermofisher). Bioinks were formed by mixing GelMA-AlgMA polymers with a final solution of 2 × 10^6^ SK-N-BE(2) or SH-SY5Y cells mL^− 1^ and in some cases 2 × 10^5^ mL^− 1^ of SW10 cells (10% of cells). Hydrogels were cultured from 2 to 10 weeks in supplemented IMDM medium replaced every 2 or 3 days.

### In vivo models

In vivo mice models were obtained as previously reported [22]. Four- to six-week-old genotyped female or male RAG1^−/−^VN^−/−^ (experimental, VN-KO) and RAG1^−/−^VN^+/+^ (wild type, VN-WT) mice were used for left adrenal gland injection of 1 × 10^6^ SK-N-BE(2) or SH-SY5Y (*n* = 40) NB cells lines (passage 0, P0). Serial tumor passages (P1 to P5) were made when tumor growth was appreciated (8 to 16 weeks) from fresh or frozen material. All experiments were carried out in accordance with the standards and care approved by the institutional animal care ethics committee (reference 2015/VSC/PEA/00083).

### Preparation of gelatin-based hydrogels

Composite hydrogels were synthetized using gelatin (denatured collagen) and alginate as previously described [39, 40]. Polymer solutions were prepared to obtain final concentrations of 5% w/v GelMA and 0, 0.5, 1, 1.5 and 2% of AlgMA. All hydrogels were fabricated using a 3D bioprinter (3DDiscovery BioSafety, regenHU, Switzerland, 365 nm, 3 W cm^ − 2^) and polymerized with UV light. Mechanical properties (Young’s module) and porosity were checked as previously reported using a Zwick Z0.5 TN instrument (Zwick-Roell, Germany) and a scanning electron microscope, respectively [39].

### Genomic analysis by high density single nucleotide polymorphism arrays (HD-SNPa)

**Fig. 1:**
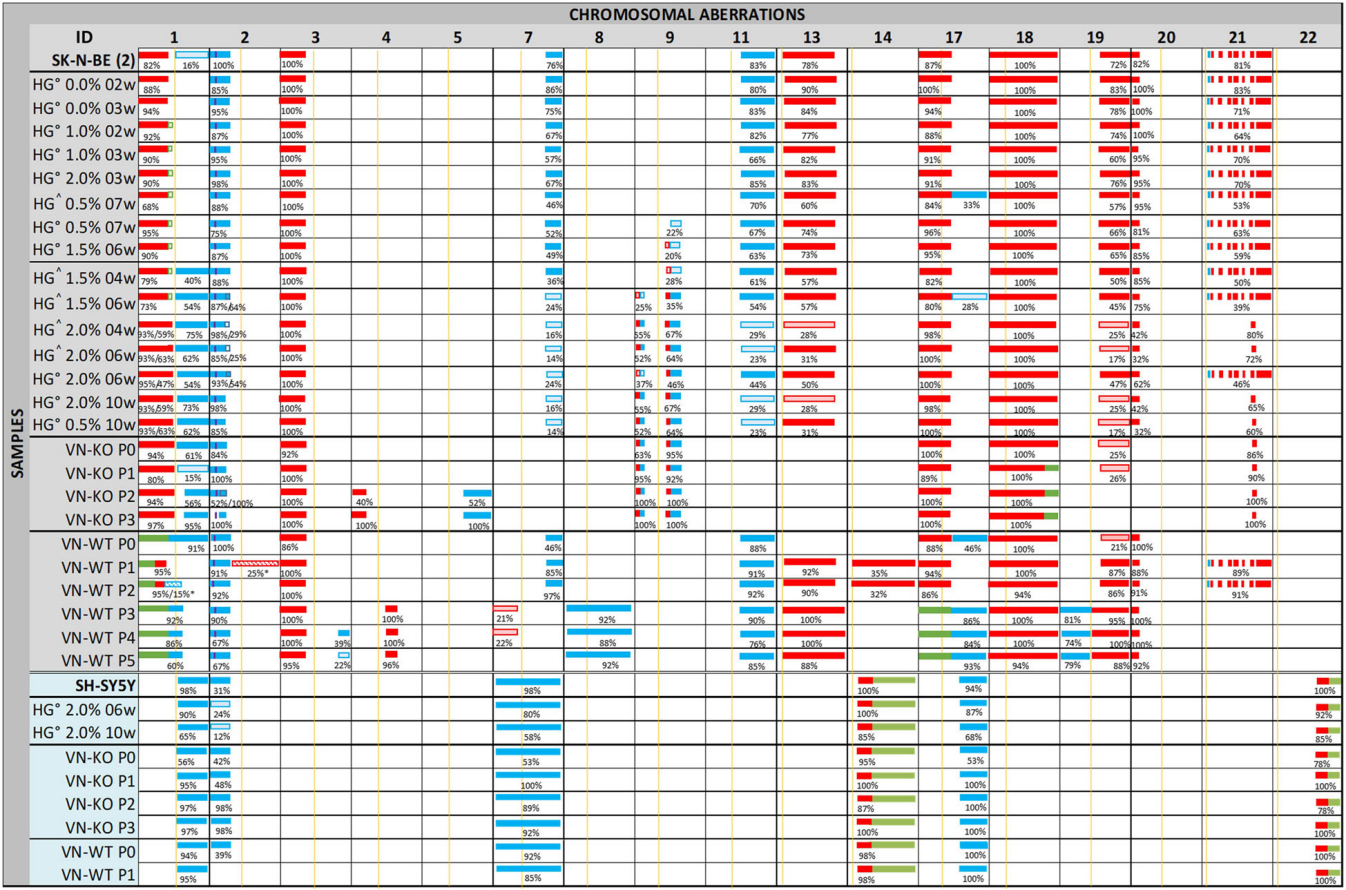
Schematic representation of the chromosomal aberrations detected by HD-SNPa in samples derived from SK-N-BE(2) and SH-SY5Y cell lines. SK-N-BE(2) and SH-SY5Y ID (identified by grey and blue columns, respectively) refer to the cell line cultured in 2D in vitro acquired from the ATCC*.* The presence of aberrations in chromosome 9 of the SK-N-BE(2) cell line grown in hydrogels alone and in co-culture with Schwann cells marks its organization within the figure. HG^°^ and HG^= neuroblasts cultured in hydrogels only and coculture with Schwann cells, respectively, followed by the percentage of AlgMA (%) and the weeks of culture (w). Tumor samples from the experimental RAG1^−/−^ VN^−/−^ mice (VN-KO) and control RAG1^−/−^VN^+/+^ mice (VN-WT) are followed by the passage number (P0-P5). The lack of symmetry of the SH-SY5Y and SK-N-BE(2) groups is due to the absence of new changes in chromosomal aberrations in samples derived from the SH-SY5Y cell line, and to the P1 growth stop of VN-WT tumors derived from this cell line. For each altered chromosome, the approximate position of centromere is marked with a yellow line. Heterozygotic gains of genomic material (3 copies) are represented in blue, heterozygotic deletions of genomic material (1 copy) are in red, and CNLOH in green. *MYCN* amplification is represented with a purple line. The percentages of cells having each chromosomal aberration according to their smooth signal are below the representative bars (e.g. when we estimated a median copy number state across a segmental chromosome aberration -SCA- of 2.62 using ChAS, we inferred the SCA affected 62% of cultured cells, and median copy number state of 1.62, implying that the deletion affected 38% of the cells in the sample [41]). When the percentage is less than 30% the background color of the aberration is lighter. The striped background and the asterisk (*) after cell percentage indicates aberrations only found in the HD-SNPa of ctDNA

DNA was extracted from 2D cultures, xenografted tumors and 3D bioprinted hydrogels using the salting out method for either frozen and paraffin-embedded tissue, as previously described [33]. Liquid biopsy study (circulating tumor DNA, ctDNA) was carried out on plasma samples with QIAamp Circulating Nucleic Acid (Qiagen) following the provided protocol. CytoScan HD was used for DNA from frozen tumors and OncoScan CNV gene chips (Affymetrix, Santa Clara, CA, USA) for DNA from paraffin-embedded tissue and plasma. DNA amplification, tagging, and hybridization to gene chips were performed according to the manufacturer’s protocol. Data were analyzed using Chromosome Analysis Suite 3.2 (ChAS) software (Affymetrix, ThermoFisher Scientific) and Nexus 10.0 Copy Number Discovery (BioDiscovery). We estimated the percentage of cells affected by each alteration (Fig. 1) with the average of the smooth signal of probes distributed throughout the whole alteration [41]. Log_2_ ratio of each aberration and aberrant cell percentage of samples grown with SK-N-BE(2) cells was estimated by TuScan tool and summarized in Table S1. To analyze which genetic pathways were most affected by the detected SCAs, we used the PANTHER tool of Gene Ontology.

### Mathematical analysis of detected genomic aberrations

All genomic aberrations detected with ChAS for SK-N-BE(2) cells were dichotomized by presence or absence in each sample. Multivariate analysis was performed using PAST software. We used the Jaccard coefficient to obtain neighbour-joining tree clustering and a matrix of similarity and distance indices.

### New generation sequencing NB-mechanopanel

We employed the SeqCap EZ HyperPlus kit from Roche Diagnostics, and the NextSeq 550 sequencer (Illumina) to obtain at least 100x coverage. Next Generation Sequencing (NGS) raw data were filtered for quality with PrintSeq and mapped against human genome GRCh38 with BWA. Mutations of mice genome were removed with two different software tools: XenofilteR and Disambiguate. Table S2 shows the NB-mechanopanel and mutations based on an allele frequency of at least 0.1 and 100x coverage.

## Results

### Chromosomal aberrations detected in 2D cell culture

**Fig. 2:**
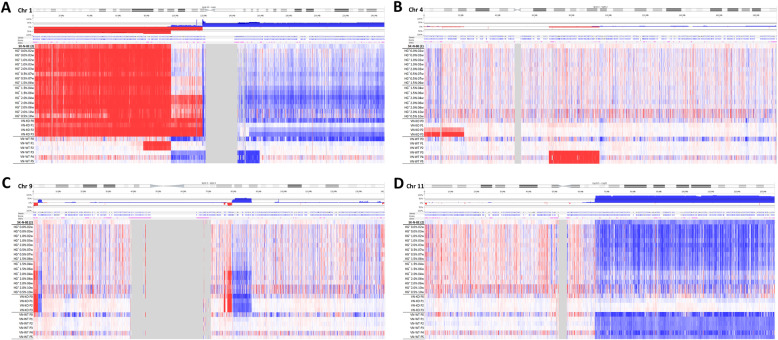
Detail of chromosomes 1 (**a**), 4 (**b**), 9 (**c**) and 11 (**d**) profiles of samples derived from SK-N-BE(2) cell line, as examples of clonal evolution. The color and intensity of each probe denotes whether it is gained (blue) or lost (red). **a** 1p-(pter-21.3) is present in all samples except those from RAG1^−/−^VN^+/+^ mice where it is substituted by CNLOH. 1p(21.3–12) is gained or lost in some samples with different percentage of affected cells. +1q(21.3-ter) is shown in many of the hydrogels in a very low cell percentage, and is clearly observed in those with high AlgMA percentage and longest culture times, and also in samples from VN-KO mice. **b** P2 and P3 tumors from VN-KO mice had a typical 4p-(ter-15.2). **c** The percentage of clones affected with FSCAs and SCA of chromosome 9 are shown in a high proportion in the stiffest and long-time culture hydrogels and in VT-KO tumors, but not in VN-WT tumors. **d** +11q(13.1-qter) was negative selected in tumors from VN-KO mice and in stiff and long culture-time hydrogels. All chromosome profile representations were obtained with Nexus 10.0 software

**Fig. 3:**
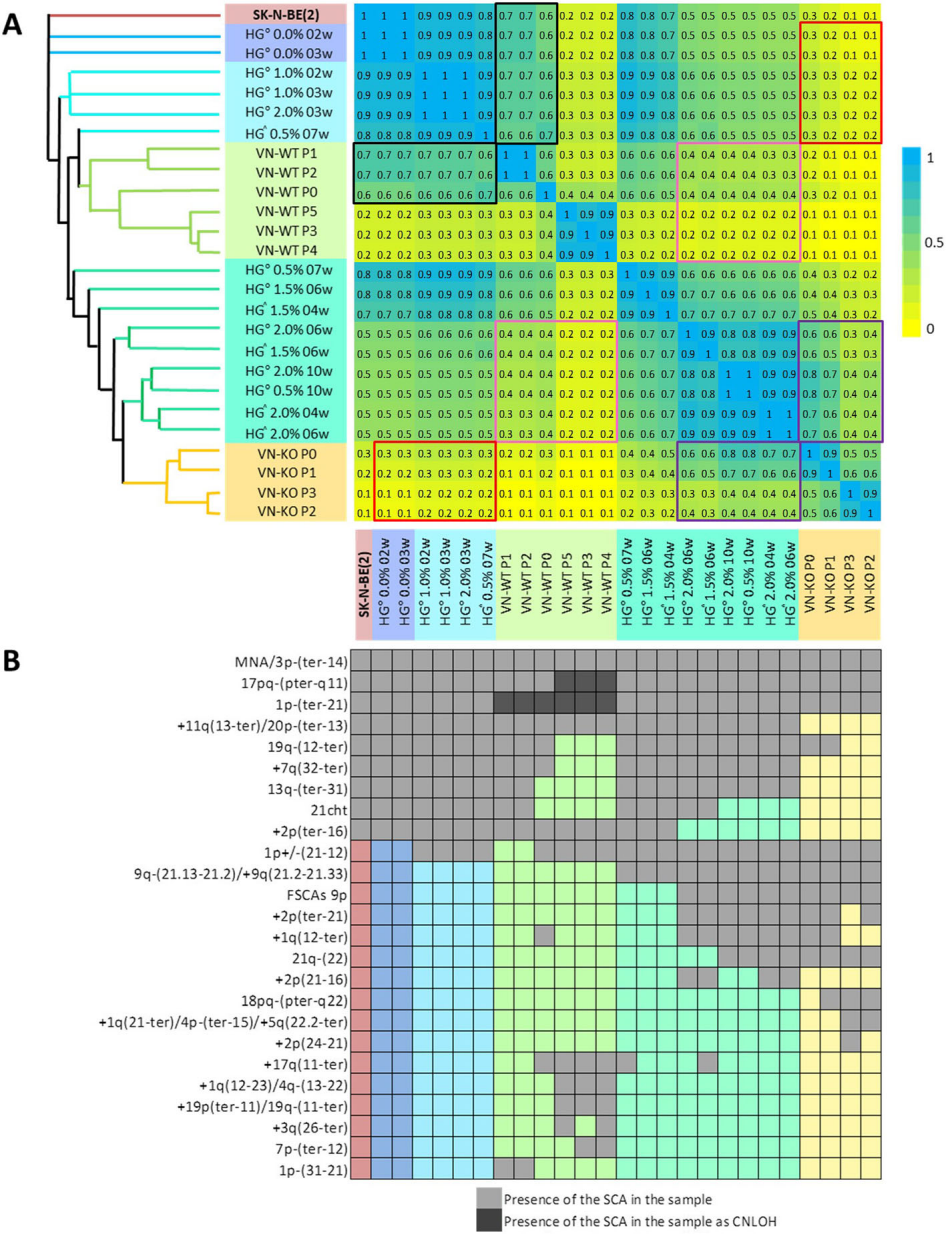
**a** Neighbor-joining tree clustering and matrix of similarity and distance indices based on the Jaccard coefficient. The highest Jaccard coefficient between samples (equal or near to 1) are represented in blue in the matrix, and with nearby branches of the same color in the tree. Lower Jaccard coefficients show a progressive fading towards yellow (equal or close to 0) and a greater distance between tree clusters. Note that softer and/or shorter cultivated hydrogels had higher similarity indices with VN-WT P0-P2 (marked in black boxes) than with VN-KO tumors (marked in red boxes) and are also nearer in the tree. Comparably, stiffer and/or longer cultivated hydrogels correlated better with VN-KO tumors (marked in purple boxes) than with VN-WT ones (marked in pink boxes), showing even smaller indices with P3 to P5. Stiffer and/or longer-time cultured hydrogels and VN-KO are clustered closer in the tree. **b** Genomic aberrations and their relationships with the models according to their presence and length, and related to the Jaccard proximity at the tree. Multivariate analysis was performed with PAST software in function to the chromosomal aberrations detected in each sample derived from the SK-N-BE(2) cell line. Presence of the SCAs in each sample is represented by light grey squares, SCAs as CNLOH by dark grey squares, and absence of genomic aberration is denoted by the remaining colors

Genomic profiling of trypsinized SK-N-BE(2) cells grown traditionally in monolayer culture revealed the presence of ten SCAs detected by HD-SNPa, as previously reported [42]: three SCAs typically observed in NB and seven SCAs classed as atypical for being less frequently present in NB. This cell line also showed a numeric chromosome alteration (NCA). As already described, SK-N-BE(2) presented genome amplification in 2p24.3 involving the *MYCN, MYCNUT, MYCNOS* and *GACAT3* genes [42, 43]. A chromothripsis-like phenomenon was also detected at chromosome 21 involving sixteen fragments. Representations of genetic findings are shown in Figs. 1, 2 and 3.

For the SH-SY5Y cell line grown in 2D culture, as previously reported [44], we found five SCAs and two atypical SCAs. We also detected a NCA and two large copy neutral loss of heterozygosity (CNLOH) (Fig. 1). Several focal segmental chromosome alterations (FSCA, < 2 Mb) and small CNLOH were also observed in both cell lines.

For SW10, we detected no chromosome aberrations.

### VN-KO with SK-N-BE(2) cells revealed SCAs similar to those of stiff hydrogels with long culture time

A description of each mice model (VN-KO and VN-WT) longer filum is shown in Figure S1.

P0 and P1 tumors grown in VN-KO mice showed, besides the 1p-(pter-21.3, 96.2 Mb) present in 2D SK-N-BE(2) cell line, a 1p-(21.3–12, 22 Mb) and a typical +1q(21.1-qter, 105 Mb), both also present in some hydrogels. Remarkably, the first fragment contained coding genes for ECM proteins like *COL11A1* and for nervous system development such as *NTNG1* and *NGF*, and the second one contained genes with important roles in cytoskeleton, like actin-related ones *(ARPC5* and *ACTN2)*, laminins (*LAMB3, LAMC1, LAMC2*), and integrin α10 (*ITGA10*). P2 and P3 tumors reduced the latter SCA to +1q(21.3-ter, 97 Mb) in the vast majority of tumor cells (Figs. 1 and 2). Like cells grown for longer times and/or stiffer hydrogels, experimental tumors P0 and P1 showed a shorter +2p(pter-21, 43 Mb) than was observed in the 2D cell line culture, which in P2 and specially in P3 became even smaller, affecting only the smallest region of overlap (SRO) +2p(24.1–21, 21 Mb). In addition, P2 and P3 had two previously undetected genomic alterations, the typical 4p-(ter-15.2, 22 Mb) and an atypical +5q(22.2-ter, 68 Mb). A very high percentage of tumor cells from all VN-KO passages showed the same chromosomal aberrations on chromosome 9: four FSCAs (9p-(24.3), +9p(24.3–24.2), 9q-(21.13), 9q-(21.13–21.2)) and a SCA (+9q(21.2–21.33) of 8 Mb, all of them also present in stiff hydrogels with long culture periods (Figs. 1 and 2). Interestingly, 9p-(24.3) contains two important genes related to migration, *KANK1* and *DOCK8*. The chromothripsis-like phenomenon was replaced by the fragment 21q-(22.13–22.2, 2.5 Mb) in all passages, as occurred in stiffest and longest-time hydrogels (Fig. 1). This fragment had a SRO with one of the altered fragments in the chromotripsis-like pattern: 21q-(22.13.22.2, 1.4 MB) (Fig. 1). The SCAs pertaining to the SK-N-BE(2) cell line in chromosomes 7, 11, 13, 19, and 20 were either absent or were only observed in proportionally few cells in tumors from VN-KO mice (Fig. 1). Finally, NCA of chromosome 18 turned into a SCA in P1 to P3 including 18pq-(pter-q22.2, 67 Mb) with the final fragment 18q(22.2-ter, 11 Mb) as a CNLOH (Fig. 1).

SK-N-BE(2) cells in VN-WT mice had high variability of SCAs between tumor passages and compared with VN-KO mice and in vitro 2D and 3D cultures. Briefly, all control mice passages changed the 1p-(pter-21.3, 96.2 Mb) for a CNLOH, maintaining only a deleted fragment 1p-(31.1–21.3, 19 Mb) in P1 and P2 tumors. The P0 sample showed a +1pq(p21.3-qter, 150 Mb), and P3 to P5 a shortest +1pq(p21.3-q23.2, 61 Mb) composed of the fragments +1p(21.3–12) and +1q(12–23). P3 to P5 tumors also had some new SCAs: 4q-(13.2–22.3), CNLOH of 17pq(pter-q11.2, 31Mb), +17q(11.2-ter), +19p(ter-11) and 19q-(11-ter). These passages had neither the +7q(32.3-qter, 28.5 Mb) nor the chromotripsis-like of chromosome 21. Finally, P3 and P4 showed 7p-(ter-12.2), and P4 and P5 had +3q(26.1-ter) in some cells (Fig. 1).

HD-SNPa detected no differences in SCAs between SH-SY5Y cells in 2D cultures or when xenografted in VN-KO mice. Interestingly, in VN-WT mice without +2p(ter-16.3, 49 Mb) the P1 tumor failed to grow in any of the eight tentative tumor xenograft attempts (Fig. S1).

To determine whether xenografted tumors had intratumoral genetic heterogeneity, we analyzed HD-SNPa of ctDNA extracted from intracardiac blood (Fig. S1). We observed high concordance between the SCAs of tumor DNA and ctDNA, except for two SCAs of VN-WT mice xenografted with SK-N-BE(2) cells, that were only present in ctDNAs (Fig. 1): i) P1 tumor had 2pq-(p16.3-qter, 196 Mb) in few clones (approximately 25%) and ii) P2 had the same +1pq(p21.3-q23.2, 61 Mb) as P3 to P5 in 15% of the clones.

### SCAs of SK-N-BE(2) cells evolved with stiffness and culture time of 3D hydrogels

Regarding in vitro 3D scaffolds cultured with SK-N-BE(2) cells, the detected SCAs changed with AlgMA concentration and culture time. Moreover, hydrogels cocultured with Schwann cells showed an enhanced positive and negative selection of some SCAs.

In detail, cells showed the same SCAs when grown for under 4 weeks in scaffoldings as when cultured in 2D, except for the new 1p- fragment (21.3–12, 22 Mb) which was found in a small percentage of cells when cultured in 1 and 2% AlgMA. The cell proportion of this SCA increased in long time hydrogels and in VN-KO mice samples (Figs. 1 and 2). Noticeably, in hydrogels with 1.5–2% of AlgMA and at least 4 weeks culturing, and hydrogel of 0.5% cultured for 10 weeks, the cell percentage of some SCAs previously observed in VN-KO mice gradually increased: i) +1q(12-qter, 105 Mb) and ii) FSCAs and SCA of chromosome 9. This positive selection was even higher in hydrogels cocultured with Schwann cells (Figs. 1 and 2). An unstable positive presence of +17q(11.2-ter, 49 Mb) was found. Finally, greater stiffness and longer time of culture 3D models, particularly those cocultured with Schwann cells, also showed clear negative selection of some SCAs, as occurred in VN-KO tumors: i) +2p(pter-16.3, 48 Mb) was gradually shortened to +2p(pter-21, 43 Mb), ii) SCAs of chromosomes 7, 11, 13, 19 and 20 showed a marked reduction of affected cells, iii) chromotripsis-like phenomenon of chromosome 21 had clonal decrease and was finally totally replaced by 21q-(22.13–22.2, 2.5 Mb).

In contrast, no genomic differences were detected by HD-SNPa between SH-SY5Y cells cultured in 2D and in 3D (Fig. 1).

### Hierarchical cluster analysis is a good tool for identifying sample similarities using genomic data

Neighbor-joining tree clustering and an array of similarity and distance indexes based on the Jaccard coefficient (Fig. 3a) were obtained, according to presence or absence of the SCAs detected with ChAS software in each sample derived from SK-N-BE(2) cells (Fig. 3b). Mathematical analyses showed the highest similarity indexes between the genomics of 2D cultured cells and hydrogels with less stiffness and/or culture time. Separated from them were tumors from the control mice, with few similarities to stiff hydrogels and experimental tumors, especially with the lower indices in P3 to P5. In general, VN-WT tumors also had low similarity between their own passages.

The genomic profiles of cells derived from the stiffer and/or longer-cultivated hydrogels, and VN-KO tumor passages were grouped together, allowing us to clearly observe the differences with the softer and/or shorter time 3D-cultures and with VN-WT tumors. Note that tumors of different VN-KO tumor passages had better similarity rates.

### Biotensegrity had impact in single nucleotide mutations

We sequenced a small customized panel (NB-mechanopanel) of specific genes, with mutations previously described in NB related to cytoskeleton remodeling and extracellular matrix changes, and other genes related to NB and cancer in general (Table S2). In SK-N-BE(2) cell line and in all samples derived from it, we identified two mutations described as p.C135F in *TP53* [45] and p.N755K in *ATRX* [46]*,* defined as pathogenic in the COSMIC database. The p.I1590 = and p.P1323L variants of *COL11A1* (in 1p), and the intronic polymorphisms c.1680-9045C > G and c.54-20738C > T of *DOCK8* (in 9p) were not detected in the majority of VN-KO tumors that had heterozygous deletions of both chromosomal regions (1p- and 9p-), despite their presence in the rest of the samples, including in the 2D cultured cell line, with allelic frequency of approximately 0.4. However, the variants p.G1504=, p.S1535P and the intronic c.652-6del of *COL11A1*, and the intronic polymorphism c.6068 + 5249A > T of *DOCK8* were maintained with the same allelic frequency (nearly 1) in all the samples.

SH-SY5Y cell lines grown in 2D, as well as in 3D hydrogels and mice, showed the pathogenic mutation described as p.F1174L in *ALK* [47], as well as p.G12V in *KRAS* not previously reported in this cell line [48]. We did not find any new significant mutations (Table S2).

### Chromosome 9 could be a hot spot for structural aberrations in HR-NB patients

**Fig. 4:**
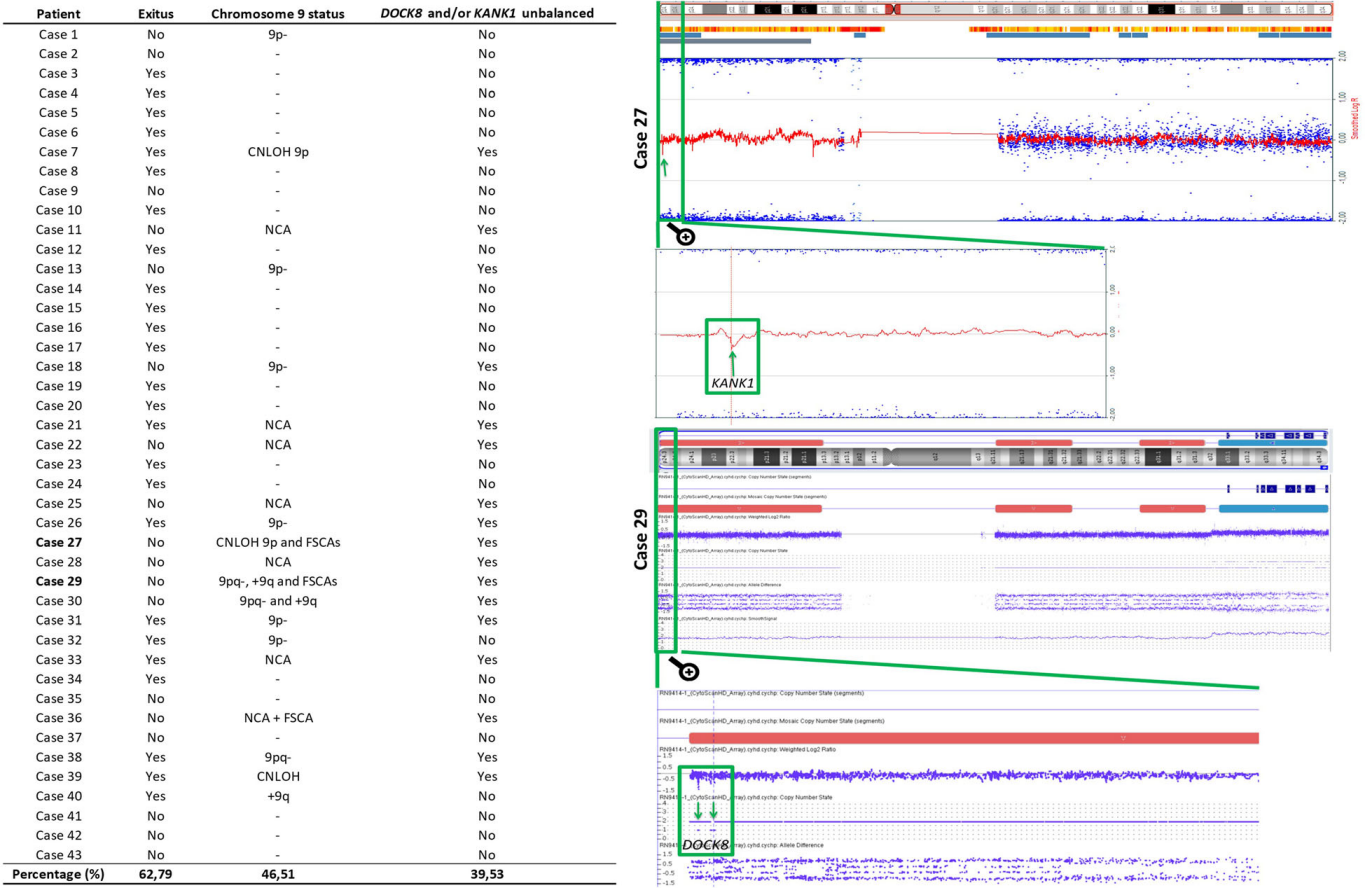
Structural aberrations detected in chromosome 9 and in genes *DOCK8* and/or *KANK1* in 43 tumor *MYCN* amplified, compared with patient survival. Detail of the chromosome 9 profile of cases 27 and 29 with focus zoomed in on both genes

We next sought to review the chromosome 9 profiles of homogeneous MNA primary tumor in HR-NB patients, due to the fact that the FSCAs and SCA of chromosome 9 were the main unusual genetic changes of the SK-N-BE(2) cells in the VN-KO tumor passages and in rigid and long-culture 3D scaffolds. Surprisingly, we observed greater than expected presence of genomic aberrations on chromosome 9 in the 43 tumor profiles checked. Specifically, 39.53% of cases had aberrations affecting the chromosomal positions of the *DOCK8* and/or *KANK1* genes (Fig. 4).

## Discussion

HR-NB is a disease with risky spatial-temporal genetic heterogeneity and high chromosome instability related to poor prognosis and therapy resistance [49, 50]. It is not yet clear whether this genomic variability could be the cause or consequence of selective evolutionary pressure in the tumor microenvironment [11]. We assumed that clonal genetic selection and/or evolution of aggressive NB cells could be promoted in part by the stiffness and composition of their surrounding matrix. To get closer to confirming this hypothesis, we selected two representative NB cell lines characterized by genomic alterations typically observed in HR-NB (MNA in SK-N-BE(2) [42] and *ALK* mutation in SH-SY5Y) [47]. Both cell lines were grown separately in hydrogels with ECM rigidity similar to the ECM of human HR-NB and xenograft tumors [39]. To make the 3D models even more biomimetic, malignant neuroblasts were cocultured with 10% Schwann cells in some cases, since even in poor stroma NB a low proportion can be found accompanying the undifferentiated or poorly differentiated neuroblasts [51]. To analyze the impact of ECM properties on genomic heterogeneity, we selected the HD-SNPa approach, complemented by the NGS technique, as one of the most commonly used procedures for detecting both MNA and SCAs in routine genetic diagnosis of NB [52]. Unlike this study, most research related to ECM tumor mechanotransduction has focused on uncovering changes in gene expression, which are difficult to translate to the clinic [53–55]. We looked for typical and/or atypical HR-NB SCA changes that could be associated with NB physics, and rapidly used for clinical translation to diagnosis and therapy. We also explored whether MNA and/or ALK-mutated cells respond similarly to ECM properties.

In previous studies, we demonstrated that elevated VN secretion by tumor cells was found in rigid ECMs, and was related to poor patient outcomes and to growth of orthotopically inoculated NB cell lines [22]. Moreover, we observed that a high amount of VN is secreted forming tracks and we postulated that VN could participate in stiffness, mediating biotensegrity and promoting tumor migration in HR-NB [22, 25]. We also reported that SK-N-BE(2) cells grown in stiff 3D hydrogels show more efficient adaptation, increased cell proliferation, high expression of the Bcl2 antiapoptosis marker, and high mRNA processing rate [39]. In the present study, genomic analysis of SK-N-BE(2) cell line revealed a number of equivalent aberrations in VN-KO tumors and in cells grown in stiffest and/or longer cultured times hydrogels. In these samples, we observed a positive selection of cells that in addition to MNA, contained: i) stable SCAs (1p-, 3p- and 17pq-); ii) SRO generated from previous SCAs (+2p and chromotripsis-like of chromosome 21) and iii) new SCAs and FSCAs (1p-, +1q, 9p-, +9p, 9q-, +9q). We also observed negative pressure which triggered progressive loss of the remaining SCAs (+7q, +11q, 13q-, 19q-, 20p-). Schwann cells seem to enhance clonal selection in NB in a more biomimetic context; however, the genomic differences found between hydrogels with vs without Schwann cells are minor and affect only the percentage of cells with some SCAs.

The evolutionary adaptation we have detected may be due to stochastic processes (generation of new aberrations and genetic drift) or also to deterministic events (clonal selection). However, the fact that cells genomes in two independent models, both with tumor spatial structures related to cellular aggressiveness, show similar genomic aberrations suggests a predominance of the deterministic evolutionary force. The increase in selection pressure, driven by adaptation to niches deliberately altered (by their lack of systemic VN, and by high stiffness and/or long 3D growth time) in both models, could prompt this similar clonal selection towards genetic aberrations detected by probably advantageous cellular phenotypes [56]. Both niches, which initially lacked VN, showed a large amount of VN secreted by neuroblasts [22]. Our results show new evidence for a probable role of VN in biotensegrity mechanotransduction by mediating the cellular response to ECM stiffness [22, 25]. Clonal evolution was faster in VN-KO tumor cells but less manageable than in hydrogels. Furthermore, cells grown in rigid 3D hydrogels showed faster selection of some SCAs and less intratumoral heterogeneity than cells with long and soft 3D growth, which could reflect a more efficient adaptation to niche [39], as occurs in aggressive patient tumors with rigid ECMs [57]. Conversely, in 3D hydrogels with short culture times, cells may not yet have been subjected to the selective process, due to their incipient adaptation to ECM. As in the early stages of patient tumors, cells in 3D bioprinted hydrogels need to grow and survive, and then adjust genetically and epigenetically to acquire the features they need to take on new roles such as migration. For this among other characteristics, 3D models are increasingly important, reducing the use of animal models and controlling the parameters focused on the study, adding degrees of complexity to gain deeper insight into the tumor microenvironment.

Interestingly, some genes of the new SCAs undergoing positive selection in VN-KO tumors and stiff and/or long-cultured hydrogels are involved in ECM composition and architecture, mechanotransduction and cell migration. In chromosome 1 there is special focus on the collagen gene *COL11A1* (in the nearer centrometic fragment of 1p-), the genes related to actin *ARPC5* and *ACTN2*, some laminins and the integrin α10 (in +1q), while the deleted FSCA of chromosome 9p contains the genes *KANK1* and *DOCK8,* both involved in actin polymerization [58–60]. In our customized NB-mechanopanel, *COL11A1* and *DOCK8* genes specifically showed certain gene variants that had disappeared in the tumors from VN-KO mice with the mentioned heterozygous deletions. The extent to which this occurred, even when the cell population with these mutations was not inconsiderable in the other growing conditions (allelic frequency: 0.4), raises the question of whether these variants might play a role in adaptation. VN of ECM could have a swift impact on the mutational profile of these migration-related genes.

Chromosome 9 aberrations have not been a focus of interest so far in HR-NB, unlike those of chromosome 1 (1p- and + 1q) [32, 61, 62]. Therefore, we decided to delve deeper into 9 chromosome abnormalities in a cohort of primary human HR-NB. Chromosome 9 aberrations involving *KANK1* and/or *DOCK8* genes were detected in 39.53% of the MNA primary HR-NB analyzed. Consequently, we recommend a spotlight on chromosome 9 aberrations [63, 64] affecting *DOCK8* and *KANK1* in NB, and especially on the sometimes denigrated FSCAs, as they could involve invasion in MNA HR-NB, as occurs in other malignant tumors [65]. The significance of these new genetic alterations found in human HR-NB, especially the frequency and prognostic impact of *DOCK8* and *KANK1*, should be determined in collaborative studies.

The SK-N-BE(2) cell line has been characterized as particularly heterogeneous [42]. Likewise, high genetic instability of xenograft mice, showing gains and losses of SCAs with passages, has already been described [56]. In all our in vitro and in vivo samples only two SCAs remained stable (with the same length and break points) in most cells: partial deletion of 3p and 17pq, adding MNA. 3p loss is a common event in HR-NB [66]; the region contains certain important suppressor genes, including *RHOA.* RhoA protein plays an significant role in mechanotransduction, as it mediates focal adhesions and stress fiber formation [67]. Its reduced signal (associated with 3p) has been linked to growth and metastasis in colorectal tumors [68, 69]. Moreover, in these tumors *RHOA* downregulation is used to match the activation of *Wnt* signal pathway and inactivation of *TGFβ*. All together they participate in initiation and progression of tumors [68]. According to Gene Onthology, the *Wnt* pathway is one of the most affected by genomic aberrations of SK-N-BE(2) cells, and most of the altered genes of these pathways are in SCAs that changed (were positive or negative selected) in VN-KO tumors and 3D bioprinted models (+7q, +9q, +11q, 13q-). *TGFβ* is located in the 19q deleted region which also had reduced presence in the samples mentioned. This gene has dual action: progression of tumors when underexpressed, and degradation of the ECM when its levels increase [70]. Meanwhile, 17pq- contains the tumor suppressor gene *TP53,* which plays an important role in tumorogenesis and aggressiveness [71]. This gene was also mutated in all samples from SK-N-BE(2) cell line with an alleleic frequency close to 1. The double mechanism involving a mutation in one of the alleles and a deletion or CNLOH of the other one has been highlighted for its combined negative impact [71]. In addition, the reported imbalance of chromosome 17, in which a loss of 17pq has been detected, is usually found with the gain of the remaining fragment of the chromosome, that is, a typical +17q [72]. 17q gain is precisely the most common genomic aberration of NB [31]. In fact, the hidden +17q became evident in some of our hydrogels and in VN-WT tumors. A probable explanation for this is that in addition to overexpression of tumor progression–promoting genes within the 17q fragments, the loss of function of genes localized on 17p- or CNLOH of 17p (such as *TP53*) may play a significant role in NB development [72].

Previously published findings could shed some light on the different impact of ECM properties on SH-SY5Y and SK-N-BE(2) cell lines when cultured in the stiffness models, much less marked on the former than the latter. PI3K/AKT is one of the most important pathways in mechanotransduction of mechanical forces, leading to gene expression and protein synthesis [73], and increased matrix stiffness has been reported to activate the PI3K/AKT signal pathway [74]. Various signaling pathways including PTEN/PI3K/AKT and RAF/MEK/ERK have been identified which control *MYCN* stabilization and act as major mediators of uncontrolled tumor growth, angiogenesis, invasion, apoptosis and cellular metabolism in NB [75–77]. An over-activated PI3K/AKT pathway could underlie the impact of ECM stiffness on SK-N-BE(2) cell line and other MNA tumor cells. Although ALK mutations signal via numerous downstream pathways, their relationship with ECM stiffness is understudied [78]. Two research groups have reported that morphological and gene expression changes in cytoskeleton and migration-related genes of SH-SY5Y cell line in stiff environments varied with the type of material [53, 54]. Moreover, Rac GTPase has been shown to regulate 3D invasion in NB lacking *MYCN* amplification [79]. It is plausible that ALK-mutated cells respond to ECM changes through epigenetic alterations, genetic mutations or post-translational modifications undetected by the techniques we employed [53, 80–82]. Various mechanisms of ECM’s influence on genomic heterogeneity have also been outlined. Some authors have suggested that in stiff solid tissues, tumor cells are squeezed (especially those that are migrating) and can suffer DNA damage, leading to new aberrations and heterogeneity [83, 84]. In this context, it been put forward that SCAs, more than mutations, could be a signature of tissue stiffness [83]. In NB, MNA and chromothripsis-like phenomenon have been described as consequences of genomic instability possibly associated with a deficient DNA repair mechanism, that could have promoted the high genetic heterogeneity observed in SK-N-BE(2) cell line in this and previous studies [42, 85, 86]. Moreover, the stiff microenvironment of the models may have prompted new chromosome breaks in SK-N-BE(2) cells with an already unstable genome. This could explain the high genomic heterogeneity found and the subsequent clonal selection of new SCAs and others already present in a minority in the cell, by leading advantageous phenotypes in the analyzed ECM stiff conditions in MNA NB cell line.

## Conclusions

Our study is further proof of the impact of biotensegrity on cancer evolution, in which dominant clones could be selected from modified sub-clones of the primary tumor by its adaptation to tumor microenvironment changes. We have described a similar genomic response of a MNA NB cell line grown in two different models (xenograft VN-KO mice and stiff hydrogels) with a niche deliberately altered in its composition and stiffness. Whether the emergence of tumor cells with genomic profiles distinct from the parental cell lines is the result of clonal selection and/or stochastic evolutionary processes should be definitively addressed by single cell DNA sequencing of the tumors. As MNA human HR-NB also showed chromosome 9 aberrations affecting the genes of interest (*DOCK8, KANK1*), functional validation as well as cell proliferation and migration studies in similar models with a greater number of HR-NB cell lines are needed for future targeted treatments. Although 3D bioprinted and in vivo models could have differences in clonal selection pressures, more accurate and biomimetic 3D scaffolds are useful to study genomics and clonal evolution of tumor cells under different stiffness conditions. A better understanding of mechanotransduction pathways related to ECM stiffness and VN involvement will expand therapeutic possibilities, and 3D bioprinted scaffolding represents an excellent tool to test the efficiency, toxicity and impact of treatment on genomics and cell heterogeneity. Furthermore, strengthened studies in a mouse model system that lead to spontaneous NB tumors recapitulating the full range of NB progression are also warranted. Finally, large collaborative studies with cells derived from primary and metastatic biopsies or tumoroids grown under different conditions, which progress in the context of a syngeneic interaction between tumor and stromal cells, are necessary to associate SCAs with the biotensegrity response of MNA and ALK-mutated NB cells.

## Supplementary information

**Additional file 1: Figure S1**: Schematic representation of the longer filums of both types of mice (experimental, vitronectin knock out [VN-KO] mice are in red boxes and control, vitronectin wild type [VN-WT] mice in grey) after orthotropic suspension inoculation of each cell line plus matrigel (1:1) or anterior pass-through tumor (fragment of about 10 mm^3^). Xenograft tumors in adrenal gland with SK-N-BE(2) cells and SH-SY5Y cell are represented in blue and green circles, respectively. For each tumor the growth time after inoculation, the volume and the percentage of neuroblasts are indicated, also specifying whether the tumor metastasized (metastasis analysis not detailed). Cases with HD-SNPa results derived from ctDNA (liquid biopsy) are reported. Note that the second passage (P2) of VN-WT mice with SH-SY5Y stopped tumor growth. (EPS 33519 kb)

**Additional file 2: Table S1:** Log_2_ Ratio and copy number of each segmental chromosome aberration -SCA-, and aberrant cell percentage of each sample from SK-N-BE(2) cell line. These parameters were estimated using ChAS software.

**Additional file 3: Table S2:** Genomic variants detected by next generation sequencing (NGS) in the sequenced samples from (A) SK-N-BE(2) cells or (B) SH-SY5Y cells, filtered by mean allelic frequencies equal to or over 0.1, and a coverage equal to or over 100x. XenofilteR and Disambiguate software were applied to remove variants from mice genome and SW10 cells. (C) Genomic regions included in NB-mechanopanel.

## Data Availability

The datasets used and analyzed in the current study are available from the corresponding author on reasonable request.

## Abbreviations

NB: Neuroblastoma
HR-NB: High-risk neuroblastoma
ECM: Extracellular matrix
VN: Vitronectin
2D: Two dimensions
3D: Three dimensions
SCAs: Segmental chromosomal aberrations
typSCA: SCA typical of NB
atypSCA: SCA atypical of NB
NCAs: Numerical chromosomal aberrations
MNA: *MYCN* amplification
CNLOH: Copy neutral loss of heterozygosity
FSCA: Focal SCA
SRO: smallest region of overlap
ctDNA: Circulating tumor DNA
GelMA: Methacrylated gelatin
AlgMA: Methacrylated alginate
VN-KO: Experimental mice RAG1^−/−^VN^−/−^
VN-WT: Control mice RAG1^−/−^VN^+/+^
P0 to P5: Serial tumor passages 0 to 5
HD-SNPa: High density single nucleotide polymorphism array
NGS: New generation sequencing
1p-(21.3–12, 22 Mb): Short arm (p) of chromosome 1 deletion (−) from cytoband 21.3 to cytoband 12 with a genomic size of 22 megabases (e.g. of structural genomic aberration)

## References

[1] Burgos-Panadero R, Lucantoni F, Gamero-Sandemetrio E, de la Cruz-Merino L, Álvaro T, Noguera R (2019). The tumour microenvironment as an integrated framework to understand cancer biology. Cancer Lett.

[2] Noguera R, Nieto OA, Tadeo I, Fariñas F, Álvaro T (2012). Extracellular matrix, biotensegrity and tumor microenvironment. An update and overview. Histol Histopathol.

[3] Aveic S, Davtalab R, Vogt M, Weber M, Buttler P, Tonini GP (2019). Calcium phosphate scaffolds with defined interconnecting channel structure provide a mimetic 3D niche for bone marrow metastasized tumor cell growth. Acta Biomater.

[4] Chen F, Zhuang X, Lin L, Yu P, Wang Y, Shi Y (2015). New horizons in tumor microenvironment biology: challenges and opportunities. BMC Med.

[5] Wullkopf L, West AKV, Leijnse N, Cox TR, Madsen CD, Oddershede LB (2018). Cancer cells’ ability to mechanically adjust to extracellular matrix stiffness correlates with their invasive potential. Mol Biol Cell.

[6] Pickup MW, Mouw JK, Weaver VM (2014). The extracellular matrix modulates the hallmarks of cancer. EMBO Rep.

[7] Zhong J, Yang Y, Liao L, Zhang C (2020). Matrix stiffness-regulated cell functions under different dimensionalities. Biomater Sci.

[8] Berger AJ, Renner CM, Hale I, Yang X, Ponik SM, Weisman PS (2020). Scaffold stiffness influences breast cancer cell invasion via EGFR-linked Mena upregulation and matrix remodeling. Matrix Biol.

[9] Filipe EC, Chitty JL, Cox TR (2018). Charting the unexplored extracellular matrix in cancer. Int J Exp Pathol.

[10] Discher DE, Janmey P, Wang YL (2005). Tissue cells feel and respond to the stiffness of their substrate. Science..

[11] Schulte M, Köster J, Rahmann S, Schramm A (2018). Cancer evolution, mutations, and clonal selection in relapse neuroblastoma. Cell Tissue Res.

[12] Greaves M (2018). Nothing in cancer makes sense except…. BMC Biol.

[13] Simi AK, Pang MF, Nelson CM (2018). Extracellular matrix stiffness exists in a feedback loop that drives tumor progression. Adv Exp Med Biol.

[14] Lampi MC, Reinhart-King CA (2018). Targeting extracellular matrix stiffness to attenuate disease: From molecular mechanisms to clinical trials. Sci. Transl. Med.

[15] Sanegre S, Lucantoni F, Burgos-Panadero R, de La Cruz-Merino L, Noguera R, Naranjo TÁ (2020). Integrating the tumor microenvironment into cancer therapy. Cancers..

[16] Rodríguez-Nogales C, Noguera R, Couvreur P, Blanco-Prieto MJ (2019). Therapeutic opportunities in neuroblastoma using nanotechnology. J Pharmacol Exp Ther.

[17] Park JR, Eggert A, Caron H (2010). Neuroblastoma: Biology, Prognosis, and Treatment. Hematol. Oncol. Clin. North Am.

[18] Tadeo I, Berbegall AP, Navarro S, Castel V, Noguera R. A stiff extracellular matrix is associated with malignancy in peripheral neuroblastic tumors. Pediatr Blood Cancer. 2017;64:e26449. 10.1002/pbc.26449 28121069

[19] Brézillon S, Untereiner V, Lovergne L, Tadeo I, Noguera R, Maquart FX (2014). Glycosaminoglycan profiling in different cell types using infrared spectroscopy and imaging. Anal Bioanal Chem.

[20] Tadeo I, Bueno G, Berbegall AP, Fernández-Carrobles MM, Castel V, García-Rojo M (2016). Vascular patterns provide therapeutic targets in aggressive neuroblastic tumors. Oncotarget..

[21] Tadeo I, Gamero-Sandemetrio E, Berbegall AP, Gironella M, Ritort F, Cañete A (2018). Lymph microvascularization as a prognostic indicator in neuroblastoma. Oncotarget..

[22] Burgos-Panadero R, Noguera I, Cañete A, Navarro S, Noguera R. Vitronectin as a molecular player of the tumor microenvironment in neuroblastoma. BMC Cancer. 2019;19:479. 10.1186/s12885-019-5693-2 PMC6532218 31117974

[23] Burgos-Panadero R, Tadeo I, Gimeno-Lluch I, Costell M, García-Rojo M, Navarro S, Noguera R. Extracellular matrix glycoproteins mechanobiology: molecular players in tumor scaffolding. Histology Histopathol. 2017;32(Supplement 1):9.

[24] Leavesley DI, Kashyap AS, Croll T, Sivaramakrishnan M, Shokoohmand A, Hollier BG (2013). Vitronectin-master controller or micromanager?. IUBMB Life.

[25] Vicente-Munuera P, Burgos-Panadero R, Noguera I, Navarro S, Noguera R, Escudero LM (2020). The topology of vitronectin: a complementary feature for neuroblastoma risk classification based on computer-aided detection. Int J Cancer.

[26] Zhu W, Li W, Yang G, Fu C, Jiang G, Hu Q (2015). Vitronectin silencing inhibits hepatocellular carcinoma in vitro and in vivo. Future Oncol.

[27] Radwan AF, Ismael OE, Fawzy A, El-Mesallamy HO (2019). Evaluation of serum integrin αvβ3 & Vitronectin in the early diagnosis of breast Cancer. Clin Lab.

[28] Ciereszko A, Dietrich MA, Słowińska M, Nynca J, Ciborowski M, Kisluk J, et al. Identification of protein changes in the blood plasma of lung cancer patients subjected to chemotherapy using a 2D-DIGE approach. PLoS One. 2019;14:e0223840. 10.1371/journal.pone.0223840 PMC6797170 31622403

[29] Heyman L, Leroy-Dudal J, Fernandes J, Seyer D, Dutoit S, Carreiras F (2010). Mesothelial vitronectin stimulates migration of ovarian cancer cells. Cell Biol Int Wiley.

[30] Schneider G, Suszynska M, Kakar S, Ratajczak MZ (2016). Vitronectin in the ascites of human ovarian carcinoma acts as a potent Chemoattractant for ovarian carcinoma: implication for metastasis by Cancer stem cells. J Cancer Stem Cell Res.

[31] Ahmed AA, Zhang L, Reddivalla N, Hetherington M (2017). Neuroblastoma in children: update on clinicopathologic and genetic prognostic factors. Pediatr Hematol Oncol.

[32] Pinto N, Mayfield JR, Raca G, Applebaum MA, Chlenski A, Sukhanova M (2016). Segmental chromosomal aberrations in localized neuroblastoma can be detected in formalin-fixed paraffin-embedded tissue samples and are associated with recurrence. Pediatr Blood Cancer.

[33] Villamón E, Berbegall AP, Piqueras M, Tadeo I, Castel V, Djos A, et al. Genetic instability and Intratumoral heterogeneity in neuroblastoma with MYCN amplification plus 11q deletion. PLoS One. 2013;8:e53740. 10.1371/journal.pone.0053740 PMC3544899 23341988

[34] Berbegall AP, Villamón E, Piqueras M, Tadeo I, Djos A, Ambros PF (2016). Comparative genetic study of intratumoral heterogenous MYCN amplified neuroblastoma versus aggressive genetic profile neuroblastic tumors. Oncogene..

[35] Uemura S, Ishida T, Thwin KKM, Yamamoto N, Tamura A, Kishimoto K, et al. Dynamics of minimal residual disease in neuroblastoma patients. Front Oncol. 2019;9:455. 10.3389/fonc.2019.00455 PMC6558004 31214500

[36] Tadeo I, Gamero-Sandemetrio E, Berbegall AP, Navarro S, Cañete A, Noguera R. 1p36 deletion results in a decrease in glycosaminoglycans which is associated with aggressiveness in neuroblastic tumors. Histol Histopathol. 2018;33:487–95. 10.14670/HH-11-947 29168879

[37] Hof KS, Bastings MMC (2017). Programmable control in extracellular matrix-mimicking polymer hydrogels. Chimia..

[38] Nolan JC, Frawley T, Tighe J, Soh H, Curtin C, Piskareva O (2020). Preclinical models for neuroblastoma: advances and challenges. Cancer Lett.

[39] Monferrer E, Martín-Vañó S, Carretero A, García-Lizarribar A, Burgos-Panadero R, Navarro S (2020). A three-dimensional bioprinted model to evaluate the effect of stiffness on neuroblastoma cell cluster dynamics and behavior. Sci Rep.

[40] García-Lizarribar A, Fernández-Garibay X, Velasco-Mallorquí F, Castaño AG, Samitier J, Ramon-Azcon J. Composite biomaterials as long-lasting scaffolds for 3D bioprinting of highly aligned muscle tissue. Macromol Biosci. 2018;18:e1800167. 10.1002/mabi.201800167 30156756

[41] Levy B, Wapner R (2018). Prenatal diagnosis by chromosomal microarray analysis. Fertil Steril.

[42] Cariati F, Borrillo F, Shankar V, Nunziato M, D’argenio V, Tomaiuolo R. Dissecting intra-tumor heterogeneity by the analysis of copy number variations in single cells: the neuroblastoma case study. Int J Mol Sci. 2019;20:893. 10.3390/ijms20040893 PMC6412524 30791380

[43] Braekeveldt N, Wigerup C, Gisselsson D, Mohlin S, Merselius M, Beckman S (2015). Neuroblastoma patient-derived orthotopic xenografts retain metastatic patterns and geno- and phenotypes of patient tumours. Int J Cancer.

[44] Kryh H, Carén H, Erichsen J, Sjöberg RM, Abrahamsson J, Kogner P (2011). Comprehensive SNP array study of frequently used neuroblastoma cell lines; copy neutral loss of heterozygosity is common in the cell lines but uncommon in primary tumors. BMC Genomics.

[45] Aminzadeh-Gohari S, Feichtinger RG, Vidali S, Locker F, Rutherford T, O’Donnel M (2017). A ketogenic diet supplemented with medium-chain triglycerides enhances the anti-tumor and anti-angiogenic efficacy of chemotherapy on neuroblastoma xenografts in a CD1-nu mouse model. Oncotarget..

[46] Lasorsa VA, Formicola D, Pignataro P, Cimmino F, Calabrese FM, Mora J (2016). Exome and deep sequencing of clinically aggressive neuroblastoma reveal somatic mutations that affect key pathways involved in cancer progression. Oncotarget..

[47] Yan B, Kuick CH, Lim M, Venkataraman K, Tennakoon C, Loh E, et al. Platform comparison for evaluation of ALK protein immunohistochemical expression, genomic copy number and hotspot mutation status in neuroblastomas. PLoS One. 2014;9:e106575. 10.1371/journal.pone.0106575 PMC4154751 25188507

[48] Osborne JK, Guerra ML, Gonzales JX, McMillan EA, Minna JD, Cobb MH (2014). NeuroD1 mediates nicotine-induced migration and invasion via regulation of the nicotinic acetylcholine receptor subunits in a subset of neural and neuroendocrine carcinomas. Mol Biol Cell.

[49] Chicard M, Colmet-Daage L, Clement N, Danzon A, Bohec M, Bernard V (2018). Whole-exome sequencing of cell-free DNA reveals temporo-spatial heterogeneity and identifies treatment-resistant clones in neuroblastoma. Clin Cancer Res.

[50] Abbasi MR, Rifatbegovic F, Brunner C, Mann G, Ziegler A, Pötschger U (2017). Impact of disseminated neuroblastoma cells on the identification of the relapse-seeding clone. Clin Cancer Res.

[51] Bourdeaut F, Ribeiro A, Paris R, Pierron G, Couturier J, Peuchmaur M (2008). In neuroblastic tumours, Schwann cells do not harbour the genetic alterations of neuroblasts but may nevertheless share the same clonal origin. Oncogene..

[52] Ambros IM, Brunner C, Abbasi R, Frech C, Ambros PF (2014). Ultra-high density HD-SNParray in neuroblastoma molecular diagnostics. Front Oncol.

[53] Li GN, Livi LL, Gourd CM, Deweerd ES, Hoffman-Kim D (2007). Genomic and morphological changes of neuroblastoma cells in response to three-dimensional matrices. Tissue Eng.

[54] Kruger TM, Bell KJ, Lansakara TI, Tivanski AV, Doorn JA, Stevens LL. A soft mechanical phenotype of SH-SY5Y neuroblastoma and primary human neurons is resilient to Oligomeric Aβ(1-42) injury. ACS Chem Neurosci. 2020;11:840–50. 10.1021/acschemneuro.9b00401 PMC7958432 32058688

[55] Yang L, Li Y, Wei Z, Chang X (1864). Coexpression network analysis identifies transcriptional modules associated with genomic alterations in neuroblastoma. Biochim Biophys Acta - Mol Basis Dis.

[56] Ben-David U, Ha G, Tseng YY, Greenwald NF, Oh C, Shih J (2017). Patient-derived xenografts undergo mouse-specific tumor evolution. Nat Genet.

[57] Tadeo I, Berbegall AP, Castel V, García-Miguel P, Callaghan R, Påhlman S, et al. Extracellular matrix composition defines an ultra-high-risk group of neuroblastoma within the high-risk patient cohort. Br J Cancer. 2016;115:480–9. 10.1038/bjc.2016.210 PMC4985353 27415013

[58] Janssen E, Tohme M, Hedayat M, Leick M, Kumari S, Ramesh N (2016). A DOCK8-WIP-WASp complex links T cell receptors to the actin cytoskeleton. J Clin Invest.

[59] Kearney CJ, Randall KL, Oliaro J (2017). DOCK8 regulates signal transduction events to control immunity. Cell Mol Immunol.

[60] Vanzo RJ, Twede H, Ho KS, Prasad A, Martin MM, South ST (2019). Clinical significance of copy number variants involving KANK1 in patients with neurodevelopmental disorders. Eur J Med Genet.

[61] Janoueix-Lerosey I, Schleiermacher G, Michels E, Mosseri V, Ribeiro A, Lequin D (2009). Overall genomic pattern is a predictor of outcome in neuroblastoma. J Clin Oncol.

[62] Khan FH, Pandian V, Ramraj S, Natarajan M, Aravindan S, Herman TS, et al. Acquired genetic alterations in tumor cells dictate the development of high-risk neuroblastoma and clinical outcomes. BMC Cancer. 2015;15:514. 10.1186/s12885-015-1463-y PMC4496850 26159519

[63] Barnhill LM, Williams RT, Cohen O, Kim Y, Batova A, Mielke JA (2014). High expression of CAI2, a9p21-embedded long noncoding RNA, contributes to advanced-stage neuroblastoma. Cancer Res.

[64] Mora J, Alaminos M, De Torres C, Illei P, Qin J, Cheung NKV (2004). Comprehensive analysis of the 9p21 region in neuroblastoma suggests a role for genes mapping to 9p21-23 in the biology of favourable stage 4 tumours. Br J Cancer.

[65] Kumps C, Fieuw A, Mestdagh P, Menten B, Lefever S, Pattyn F (2013). Focal DNA copy number changes in neuroblastoma target MYCN regulated genes. PLoS One.

[66] Pugh TJ, Morozova O, Attiyeh EF, Asgharzadeh S, Wei JS, Auclair D, et al. The genetic landscape of high-risk neuroblastoma. Nat Genet. 2013;45:279–84. 10.1038/ng.2529 PMC3682833 23334666

[67] Liu H, Liu Y, Zhang X, Wang X. Current study of RhoA and associated signaling pathways in gastric cancer. Curr Stem Cell Res Ther. 2020;15:607–13. 10.2174/1574888X15666200330143958 32223738

[68] Dopeso H, Rodrigues P, Bilic J, Bazzocco S, Cartón-García F, Macaya I (2018). Mechanisms of inactivation of the tumour suppressor gene RHOA in colorectal cancer. Br J Cancer.

[69] Dyberg C, Fransson S, Andonova T, Sveinbjörnsson B, Lännerholm-Palm J, Olsen TK (2017). Rho-associated kinase is a therapeutic target in neuroblastoma. Proc Natl Acad Sci U S A.

[70] Lebrun J-J. The dual role of TGFβ in human Cancer: from tumor suppression to Cancer metastasis. ISRN Mol Biol. 2012;2012:381428. 10.5402/2012/381428 PMC4899619 27340590

[71] Liu Y, Chen C, Xu Z, Scuoppo C, Rillahan CD, Gao J, et al. Deletions linked to TP53 loss drive cancer through p53-independent mechanisms. Nature. 2016;531:471–5. 10.1038/nature17157 PMC4836395 26982726

[72] Schleiermacher G, Raynal V, Janoueix-Lerosey I, Combaret V, Aurias A, Delattre O (2004). Variety and complexity of chromosome 17 translocations in neuroblastoma. Genes Chromosom Cancer.

[73] Yano H, Choudhury ME, Islam A, Kobayashi K, Tanaka J (2015). Cellular mechanotransduction of physical force and organ response to exercise-induced mechanical stimuli. J Phys Fit Sport Med.

[74] Gao X, Qiao X, Xing X, Huang J, Qian J, Wang Y (2020). Matrix stiffness-Upregulated MicroRNA-17-5p attenuates the intervention effects of metformin on HCC invasion and metastasis by targeting the PTEN/PI3K/Akt pathway. Front Oncol.

[75] Joshi S (2020). Targeting the tumor microenvironment in neuroblastoma: recent advances and future directions. Cancers..

[76] Cage TA, Chanthery Y, Chesler L, Grimmer M, Knight Z, Shokat K (2015). Downregulation of MYCN through PI3K inhibition in mouse models of pediatric neural cancer. Front Oncol.

[77] Petrov I, Suntsova M, Ilnitskaya E, Roumiantsev S, Sorokin M, Garazha A, et al. Gene expression and molecular pathway activation signatures of MYCN-amplified neuroblastomas. Oncotarget. 2017;8:83768–80. 10.18632/oncotarget.19662 PMC5663553 29137381

[78] Hallberg B, Palmer RH. The role of the ALK receptor in cancer biology. Ann. Oncol. 2016;27(Supplement 3):iii4–15. 10.1093/annonc/mdw301 27573755

[79] Mitchell CB, O’Neill GM. Rac GTPase regulation of 3D invasion in neuroblastomas lacking MYCN amplification. Cell Adhes Migr. 2017;11:68–79. 10.1080/19336918.2016.1183868 PMC5308223 27224546

[80] Dong SY, Guo YJ, Feng Y, Cui XX, Kuo SH, Liu T (2016). The epigenetic regulation of HIF-1α by SIRT1 in MPP+ treated SH-SY5Y cells. Biochem Biophys Res Commun.

[81] Gu X, Sun J, Li S, Wu X, Li L (2013). Oxidative stress induces DNA demethylation and histone acetylation in SH-SY5Y cells: potential epigenetic mechanisms in gene transcription in Aβ production. Neurobiol Aging.

[82] Ferrari S, Pesce M (2019). Cell-based mechanosensation, epigenetics, and non-coding RNAs in progression of cardiac fibrosis. Int J Mol Sci.

[83] Pfeifer CR, Alvey CM, Irianto J, Discher DE (2017). Genome variation across cancers scales with tissue stiffness-an invasion-mutation mechanism and implications for immune cell infiltration. Curr Opin Syst Biol.

[84] Xia Y, Pfeifer CR, Zhu K, Irianto J, Liu D, Pannell K (2019). Rescue of DNA damage after constricted migration reveals a mechano-regulated threshold for cell cycle. J Cell Biol.

[85] Ratnaparkhe M, Wong JKL, Wei PC, Hlevnjak M, Kolb T, Simovic M (2018). Defective DNA damage repair leads to frequent catastrophic genomic events in murine and human tumors. Nat Commun.

[86] Schwab M, Corvi R, Amler LC (1995). N-MYC oncogene amplification: a consequence of genomic instability in human neuroblastoma. Neurosci..

